# Cardiovascular Toxicities of Radiation Therapy and Recommended Screening and Surveillance

**DOI:** 10.3390/jcdd10110447

**Published:** 2023-10-31

**Authors:** Gabriela Narowska, Sakshi Gandhi, Allison Tzeng, Eman A. Hamad

**Affiliations:** Department of Cardiology, Temple University Hospital, Philadelphia, PA 19140, USA; gabriela.narowska@tuhs.temple.edu (G.N.); allison.tzeng@tuhs.temple.edu (A.T.)

**Keywords:** radiation, cardiotoxicity, screening, prevention, surveillance, constrictive pericarditis, valvular disease, coronary artery disease

## Abstract

Radiation therapy is a key part of treatment for many cancers. Vast advancements in the field of radiation oncology have led to a decrease in malignancy-related mortality, which has uncovered some of the long-term side effects of radiation therapy. Specifically, there has been an increase in research looking into the cardiovascular side effects of chest radiation therapy for cancers of the esophagus, breast, and lung tissue as well as lymphomas. The manifestations of cardiac injury from irradiation range from short-term complications, such as pericarditis, to long-term damage including cardiomyopathy, valvular disease, and conduction disturbances. The aims of this article are to describe the cardiovascular side effects and the associated risk factors, to discuss risk reduction strategies, and to provide guidance in pre-radiation screening, post-radiation surveillance, and the management of these conditions.

## 1. Introduction

Vast advancements in cancer-related therapies have been associated with a decline in malignancy-related mortality, leading to a significant increase in the number of survivors. This has brought greater recognition to the adverse effects of cancer therapies, specifically the cardiovascular toxicity profile. Cardiovascular disease is the leading cause of non-malignancy-related deaths in cancer survivors and is known to be related to the amount of chest irradiation patients undergo [1]. For instance, patients who have undergone irradiation for breast cancer have a 3.1% increased risk of cardiac death per each Gray unit of thoracic radiation [2]. Nonetheless, radiation therapy plays a significant role in the treatment of cancer and is used on more than 50% of patients diagnosed with cancer [2]. The benefits of radiation therapy can be curative, adjunctive, and/or palliative. Cardiovascular toxicities, including coronary artery disease (CAD), valvular heart disease, heart failure (HF), myocarditis, cardiomyopathies, arrhythmias, and pericardial syndromes are some of the known sequelae of chest irradiation. As it is often used for the treatment of lymphomas and cancers of the esophagus, breast, and lung tissues, these are the most common cancers that expose the heart to irradiation [1,2]. Similarly, pre-existing cardiovascular disease can complicate radiation treatment. There are many articles outlining proposed guidelines for the cardiovascular screening of patients undergoing radiation, however, there is no consensus on the optimal surveillance approach for these patients. In this paper, we aim to summarize the many reported mechanisms of cardiotoxicity related to radiation exposure in cancer and the guidelines for the management of cardiovascular screening and surveillance.

## 2. Radiation Therapy

Radiation therapy involves the use of X-rays, gamma rays, or charged particles as an agent to destroy cancer cells. As these electrically charged particles pass through tissues, they deposit high amounts of energy that directly damage the deoxyribonucleic acid (DNA) of cells, thereby blocking their proliferative abilities [3]. Radiation can also indirectly cause cell death through the creation of free radicals that can, in turn, lead to DNA damage [3]. As the energy passes through tissues, it is not able to distinguish cancer cells from nearby healthy cells. The goal of therapy is to kill malignant cells while minimizing the exposure of healthy cells to radiation by utilizing various strategies, such as patient positioning, beam direction, 3D radiation therapy, and image-guided radiotherapy. Radiation can be used in isolation or in conjunction with other treatment modalities such as chemotherapy, immunotherapy, or surgery.

## 3. Factors Associated with Increased Risk of Cardiotoxicity

**Figure 1:**
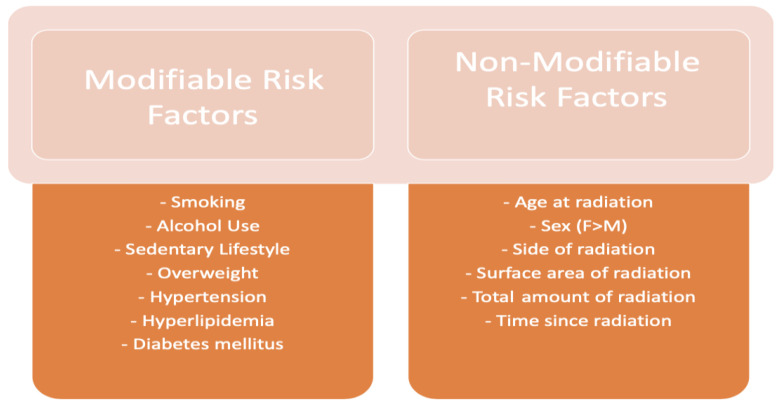
Risk factors associated with an increased risk of cardiovascular complications of mediastinal radiation.

Several risk factors have been described to increase the likelihood of radiation-induced cardiovascular disease. The total dose of radiation, measured in Gray units, has been identified as the most significant risk factor for developing cardiovascular disease (Figure 1). There is no clear value of Gray units at which the risk significantly increases and complications can be seen with any dose. However, several studies have shown that a total radiation dose above 30 Gray units has a linear association with an increased risk of valvular heart disease [4]. Similarly, a trial looking at the dose-escalation of radiation therapy for lung cancer demonstrated an increased risk of subsequent cardiac events [4]. A study by Darby et al. found that the rates of major coronary events increased linearly by 6.4% with each Gray unit of radiation. The location of radiation also plays a significant role. Women with radiation to the left breast had higher incidences of complications compared to those with irradiation to the right breast alone [5]. Other risk factors include the volume of heart irradiated, the extent of coronary artery involvement, and the concomitant use of cardiotoxic chemotherapy agents, such as anthracyclines, trastuzumab, and fluorouracil [4].

In addition to the timing, dosing, and the type of radiation used, there are also pre-existing conditions that increase patients’ risk of developing complications. Patients with pre-existing hyperlipidemia, diabetes, coronary artery disease (CAD), inactivity, and tobacco and alcohol use are at an increased risk compared to patients without pre-existing conditions [5]. Patients who undergo radiation at a younger age are more likely to develop cardiotoxicity (Figure 1) [5]. Women are also at an increased risk compared to men, which has been attributed to postmenopausal changes leading to the loss of the cardioprotective effects of estrogen [6].

## 4. Cardiovascular Complications of Radiation Therapy

The cardiovascular complications related to radiation therapy may be immediate and short-term, while other complications are delayed with long-term sequelae. The short-term complications are related to inflammatory changes in the affected tissue, such as pericarditis or myocarditis [7]. In the long term, the affected tissues become tough and fibrotic, leading to functional issues such as coronary artery disease, valvular dysfunction, heart failure, and damage to the electrical conduction system leading to arrhythmias [7].

## 5. Adverse Cardiovascular Effects: Pathophysiology and Management

### 5.1. Pericardial Disease

#### 5.1.1. Incidence and Pathophysiology

Pericardial disease is a very common manifestation of radiation-induced heart disease. It can range in severity from asymptomatic pericardial calcifications to diffuse constrictive pericarditis and cardiac tamponade. One study found that out of 167 patients who underwent radiation for primary esophageal cancer, pericardial effusions developed in 35.9% of patients. Of these, 8.4% had symptomatic effusions requiring treatment. The average pericardial doses of radiation were significantly higher in patients who developed symptomatic pericardial disease [8]. A smaller study of autopsy reports in patients with a history of chest radiation found that 70% of patients had evidence of pericardial disease ranging from effusion to constrictive pericarditis [9]. High doses of radiation to the mediastinum can cause inflammation in the pericardium [4,9]. Over time, as the immune system responds to the localized inflammation, the adipose tissue of the pericardium is replaced with collagen and fibrin, resulting in fibrosis and calcification. The distribution of pericardial disease is highly dependent on the proportion of the heart that was within the irradiation field [4,9]. Fibrosis also impairs venous drainage of the pericardial space, which can present as either acute or chronic pericardial effusion, and in some cases, cardiac tamponade [10].

Given that a large portion of patients with pericardial disease are asymptomatic, it is thought that the total number of patients with radiation-induced pericardial disease is under-reported [10]. Acute pericarditis and pericardial effusions are generally seen in conjunction with very high doses of radiation, like those administered in cases of Hodgkin’s lymphoma or esophageal cancer. Most cases of pericardial disease tend to present 5–10 years after radiation exposure [10].

#### 5.1.2. Management

The management of pericardial disease depends on the acuity and presence of symptoms. For pericardial effusions that were incidentally found and if the patient is hemodynamically stable and asymptomatic, close monitoring is recommended. For patients who are symptomatic, pericardiocentesis should be pursued. In cases of cardiac tamponade, pericardiocentesis is emergent.

The management of radiation-induced acute pericarditis is similar to that of other causes of pericarditis. The standard recommended treatment is nonsteroidal anti-inflammatory agents (NSAIDs). There are not many studies that look at the use of colchicine in these patients, however, it has been shown to be effective in some cases [11]. The use of steroids has also not been well-established and should be reserved for the management of hemodynamically significant pericarditis in the acute time frame post-radiation [12]. For resistant or recurrent pericarditis, beta receptor antagonists can be used [12]. A proposed mechanism by which beta-blockers can reduce recurrence and assist with symptom management is related to the reduction of pericardial layer friction by decreasing heart rate [13].

The literature shows that 20% of patients with acute pericarditis will develop chronic constrictive pericarditis [14]. Radiation-induced constrictive pericarditis has very unfavorable outcomes. The treatment options for these patients are often limited to either a partial or total pericardiectomy. In a study looking at surgical outcomes in patients undergoing surgical interventions for radiation-induced constrictive pericarditis, the 5-year survival rate was only 51%, compared to 83% for non-radiation-related diseases [15]. Another study of 163 patients who underwent pericardial stripping found a 7-year survival rate of only 27% [16].

### 5.2. Coronary Artery Disease

#### 5.2.1. Incidence and Pathophysiology

Coronary artery disease (CAD), including acute coronary syndrome and myocardial infarction, is the most common manifestation of radiation-induced cardiovascular disease. It has been widely studied in patients with a history of thoracic radiation treatment for Hodgkin’s Lymphoma, and it is estimated that up to 85% of patients who underwent treatment went on to develop coronary artery disease [7]. Another retrospective study of 415 patients who underwent radiation therapy for the treatment of Hodgkin’s Lymphoma noted that the mean time leading to the development of coronary artery disease was 9 years [17]. Additionally, a randomized trial of breast cancer patients comparing those who underwent radiation in addition to surgery and those who underwent surgery alone noted a significantly higher mortality rate due to coronary artery disease in the post-radiation therapy group, and that the relative risk of the development of CAD appeared to be linearly proportional to the amount of radiation the heart received [5]. Patients with a prior history of radiation therapy also have a 2.5 times increased risk of fatal MI as compared to that of the general population [18].

High doses of radiation result in inflammation and endothelial injury of the coronary vasculature. This precipitates cytokine release which leads to further damage and remodeling, resulting in accelerated stenosis and atherosclerosis when compared to the general population.

#### 5.2.2. Management

The management of coronary artery disease in this population necessitates increased vigilance and screening. Percutaneous interventions are preferred over coronary artery bypass graft (CABG) surgeries given the post-radiation changes that affect other elements of the thoracic space, leading to increased postoperative wound complications. In patients with a history of thoracic radiation treatment, the usual arterial and venous conduits are often fibrotic and stenosed as well, making them poor choices for grafts. Additionally, thoracic radiation results in scarring and fibrosis of the skin and subcutaneous tissues, which puts this population at higher risk for bleeding complications and poor wound healing. A study in 2013 reported an overall survival rate of 45%, at a mean of 7.8 years after CABG, for patients who underwent mediastinal radiation [19]. This population also tends to have significant atherosclerosis and calcification of the thoracic aorta, which significantly increases the risk of stroke when placed on cardiopulmonary bypass [19]. Several studies have demonstrated poor outcomes and worse mortality post-CABG in this population, which have been attributed to the extent of radiation-induced fibrosis and scarring in the mediastinum, and as a result, percutaneous interventions are preferred [20].

### 5.3. Cardiomyopathy

#### 5.3.1. Incidence and Pathophysiology

Radiation therapy that involves the thorax significantly increases the risk of nonischemic cardiomyopathy. Cardiomyopathies are estimated to affect about 10% of patients who undergo radiation therapy [4]. Several mechanisms for this have been described, including restrictive physiology related to pericarditis and fibrotic changes caused by radiation. High doses and large fields of radiation are associated with inflammation, injury, and death of the myocardial cells. As a result, the myocardium is infiltrated by myofibroblasts which leads to an increase in collagen deposition [3,4]. The myocardium becomes stiff and fibrosed, resulting in a restrictive cardiomyopathy with diastolic dysfunction [3,4]. Radiation can also result in endothelial injury to the coronary vasculature, leading to poor oxygenation in the myocardial tissue. Over time, this leads to myocyte death and myofibroblast proliferation which also results in collagen deposition and fibrosis.

#### 5.3.2. Management

Nonischemic cardiomyopathy secondary to thoracic radiation presents with the same symptomatology as ischemic cardiomyopathy, including dyspnea on exertion, lower extremity edema, orthopnea, and exercise intolerance. Heart failure symptoms are managed with standard goal-directed medical therapy including beta-blockers, angiotensin-converting enzyme inhibitors, angiotensin receptor blockers, neprilysin inhibitors, and sodium-glucose cotransporter-2 inhibitors [21]. The data are lacking in evaluating the mortality benefit of GDMT in this population compared to other etiologies of cardiomyopathy, such as ischemic or hypertensive [21]. In advanced stages of radiation-induced cardiomyopathy, heart transplant remains the main-stay of life-prolonging therapy. However, the 5-year survival rate in these patients is lower compared to the cardiomyopathy of other etiologies (58% vs. 73%), which has been attributed to sternal wound complications and postoperative respiratory and renal failure [10,22].

In addition to these therapies, many nutraceuticals have been shown to reduce cardiotoxicity in clinical models, although current guidelines do not mention the use of nutraceuticals in the management of cardiotoxicities. Quercetin has been shown to protect radiation-induced DNA from damage and apoptosis in the kidney and bladder tissues of rats [22]. In a more recent study, polydatin was shown to reduce cardiotoxicity by decreasing pro-oxidative stress and pro-inflammatory cytokines [23].

### 5.4. Valvular Disease

#### 5.4.1. Incidence and Pathophysiology

Thoracic radiation poses a significant risk for developing valvular heart disease in the long term. Studies have demonstrated that the risk increases significantly in patients who receive total radiation doses of >30 Gy units. A study evaluating the 30-year cumulative risk of valvular heart disease in patients undergoing thoracic radiation found that patients receiving a total radiation dose of <30 Gy had a cumulative risk of 3% compared to 12.4% for those receiving >40 Gy [24]. Similarly, another study demonstrated a 45% prevalence of valvular disease in 15-year lymphoma survivors who received a total radiation dose >30 Gy, marking it as an independent risk factor for valvular heart disease [25]. The pathophysiology of valvular disease after radiation is associated with fibrosis and the thickening of valve leaflets. Left-sided valves are more commonly affected, specifically the aortic valve. Additionally, regurgitation is seen more commonly than stenosis, which is thought to be secondary to the effects of higher-pressure circulation on damaged valves [26]. Pathology studies of excised valves demonstrated increased collagen and decreased calcified tissue when compared to patients who did not have prior radiation exposure. On echocardiography, radiation-induced valvular damage characteristically leads to diffuse valvular thickening, calcification, and fibrosis of the aortic apparatus including the root, leaflets, and annulus. It may also affect the mitral valve annulus and leaflets [3].

In addition to aortic valve disease, patients are also at risk of developing mitral valve disease. The mitral valve annulus and leaflets are primarily affected, characteristically including the middle and base rather than the tips and commissures, which could lead to underestimates of the severity of the stenosis [26]. Right-sided valvular disease occurs with less frequency than left-sided valvular disease owing to less exposure to the adverse effects of radiation [26]. While tricuspid valvular disease is a known phenomenon, there are no known studies published that describe the frequency of tricuspid valvular disease or the efficacy of treatment. This is an area that requires further investigation and research.

#### 5.4.2. Management

The management of valvular disease includes both surgical and transcatheter approaches. Notably, surgical valve replacement procedures in post-chemoradiation patients can be particularly difficult due to the presence of chest wall scarring and the possibility of delayed wound healing in the setting of radiation. The 2020 ACC/AHA guidelines for valvular heart disease include prior radiation therapy as a factor associated with prohibitive surgical risk for patients with severe symptomatic aortic stenosis [27]. Several studies demonstrate the increased mortality risk for patients who undergo surgical procedures after radiation therapy [19].

For aortic valve replacement, the transcatheter approach has been demonstrated in numerous studies to have superior outcomes in comparison to surgical repair. An observational study in 2019 of patients with severe aortic stenosis and a history of chest irradiation found that patients who underwent transcatheter aortic valve repair (TAVR) had lower 30-day and 1-year mortality rates than with surgical aortic valve repair (SAVR), fewer episodes of postoperative atrial fibrillation, and shorter lengths of hospital stays [27]. A year later, a larger study of patients who had undergone mediastinal radiation found TAVR was associated with lower in-hospital mortality in comparison to SAVR (1.2% vs. 2.0%, adjusted odds ratio: 0.27; *p* < 0.02). This study also demonstrated TAVR to be associated with lower rates of acute kidney injury, use of mechanical circulatory support, bleeding and respiratory complications, and shorter length of hospital stays [28].

The consensus of available data demonstrates TAVR to be the superior alternative to SAVR in patients with radiation-induced aortic valvular disease. Given the increased surgical risk for patients who have undergone radiation therapy, TAVR should be considered the preferred treatment method for aortic valve repair as it is associated with lower mortality rates, shorter length of hospital stays, and fewer postoperative complications [10,25,26].

There are limited studies on the effects of mitral valve intervention and its outcomes. A small study exists, including 15 patients, that found transcatheter edge-to-edge repair with MitraClip therapy was associated with improvement in both six- and twelve-month follow-up along with a reduction in mitral valve regurgitation, suggesting that the transcatheter approach may be beneficial. However, it should be noted that hemodynamic mitral valve stenosis developed in three of the fifteen patients, which further highlights the progressive nature of radiation valvulopathy [28].

### 5.5. Conduction System Disease

#### 5.5.1. Incidence and Pathophysiology

Conduction system disease can result from a variety of mechanisms related to radiation. Radiation can result in direct inflammation and injury of the conduction system, or via fibrosis and ischemia of the surrounding myocardial tissue. ECG abnormalities were seen in up to 75% of patients who received chest radiation with a total dose of >40 Gy units [29]. Transient and asymptomatic arrhythmias may be seen briefly within the first year of therapy and usually are harmless, however permanent damage to the conduction system usually presents 10 years after RT completion [30]. It can present in a variety of ways, depending on which part of the conduction system is affected. Patients can have prolonged QT intervals, sinus node or atrioventricular node dysfunction, fascicular blocks, or even ventricular tachycardias. Right bundle branch blocks are more common than left bundle branch blocks because the right-sided conduction system is anterior and more likely to be exposed to radiation [30]. Conduction system disease is estimated to affect 4–5% of patients with a history of radiation therapy, and often presents in conjunction with other types of radiation-induced heart diseases like cardiomyopathy and coronary artery disease [7].

#### 5.5.2. Management

Patients who develop symptoms or evidence of conduction disease warrant the same workup as individuals without a history of radiation therapy. Patients should be referred to an electrophysiologist and ambulatory cardiac monitoring should be performed when arrhythmias are suspected. The management of conduction system disease, similarly to that of the general population, is dependent on the manifestation, and ranges from monitoring and receiving antiarrhythmic agents to device implantations or ablations.

## 6. Risk Assessment and Pre-Radiation Optimization

### 6.1. Risk Assessment

In addition to the risk caused by the radiation itself, the patient’s pre-existing cardiovascular risk factors can lead to a cumulative hazard for the development of cardiovascular complications. Some of these risk factors are non-modifiable such as age, sex, and the location and size of the mass. Other risk factors, however, are modifiable and should be optimized prior to treatment. These include hypertension, hyperlipidemia, diabetes mellitus, tobacco and alcohol use, obesity, and a sedentary lifestyle (Figure 1). It is important to identify pre-existing risk factors for cardiovascular disease prior to initiating radiation therapy. A thorough history should be gathered and a focused physical exam should be performed, including an auscultation for murmurs and an evaluation for lower extremity edema and jugular venous distention. At this pre-screening visit, laboratory studies should include a lipid panel and a hemoglobin A1c, as hyperlipidemia and diabetes mellitus are both linked with an increased risk of cardiovascular disease. Additionally, each patient should have an electrocardiogram (ECG) and transthoracic echocardiogram (TTE) prior to radiation therapy.

### 6.2. Optimization of Pre-Existing Conditions

Current cardiovascular disease prevention guidelines do not include radiation therapy as a risk modifier. Therefore, we recommend using the current literature on cardiovascular risk reduction for the general population. For patients with pre-existing hypertension, we recommend initiating antihypertensives with a blood pressure goal of <130/80 mmHg, which is in line with the 2017 ACC/AHA Hypertension Guidelines and the 2023 ESH Guidelines [31,32]. In accordance with the 2019 ACC/AHA Primary ASCVD Prevention Guidelines, we recommend that patients with hyperlipidemia should be further risk stratified using the Atherosclerotic Cardiovascular Disease (ASCVD) Risk Assessment [33]. Patients with a score >7.5% should be started on statin therapy with a goal of an LDL <70 mg/dl [33]. For patients with an ASCVD <7.5%, coronary artery calcium (CAC) scoring should be considered to better assess the extent of atherosclerosis. For patients with a CAC score of 0, it is reasonable to pursue lifestyle modifications and reassess the need for statin therapy in 5–10 years [33]. For patients with a CAC score between 1 and 99, initiation of statin therapy should be considered, especially for patients >55 years of age [33]. For patients with a CAC of 100 or higher, we recommend starting a statin and low-dose aspirin [33]. Diabetes mellitus should be treated aggressively with a goal hemoglobin A1c of <7 mg/dl, according to the 2010 ADA Standards of Medical Care [34]. Hemoglobin A1c should be monitored every 3–6 months and maintained at the goal for the duration of therapy and thereafter.

### 6.3. Optimization of Radiation Administration

Minimizing cardiac exposure to radiation is the best risk reduction strategy. A personalized approach should be applied for each patient with an effort to achieve the desired radiation goal using the smallest total dose and frequency of therapies required. Studies have shown that radiation-induced cardiotoxicity is dose-dependent, especially when total doses exceed 30 Gy units [35]. Additionally, a multicenter study demonstrated that the fractionated daily dose increases the risk of pericardial effusions in patients who underwent radiation therapy for esophageal cancer [36]. The average recommended daily dose is smaller than 2 Gy [37]. In addition to dose adjustments, new techniques have evolved to limit cardiac exposure. At many institutions, the standard radiation technique is the three-dimensional conformal RT (3D CRT) which uses photon beams to damage cells in the affected area. Some institutions are using intensity-modulated RT (IMRT), which is a newer modality that delivers higher doses of RT to tumor cells while reducing the dose of Gy units delivered to selected normal tissues. An even newer radiation modality, proton beam therapy (PBT), is an advanced modality that precisely delivers a beam of protons, instead of photons, to destroy tumor cells. Compared with traditional radiation, protons lose energy very rapidly as they penetrate through tissues, which results in a precisely localized peak in dose. A recent randomized phase IIB trial compared IMRT with PBT to evaluate the total toxicity burden and progression-free survival for patients undergoing the treatment of advanced esophageal cancer. The trial was stopped early due to an interim analysis showing the significant superiority of PBT compared to IMRT [38]. Respiratory motion management has been shown as a successful risk reduction strategy. The SAVE-HEART study used the deep inspiration breath-hold in patients undergoing left-sided breast radiation and found this method to reduce the mean cardiac radiation dose by 35% compared to normal breathing [39]. Additionally, in a large study of 2,232 patients who underwent thoracic radiation for Hodgkin’s lymphoma, subcarinal blocking was found to reduce the relative risk of cardiac disease from 5.3 (CI: 3.1–7.5) to 1.4 (CI: 0.6–2.9) [40].

## 7. Surveillance and Follow-Up

Patients with a prior history of radiation should be monitored closely for symptoms and signs of radiation-induced cardiovascular disease. As described above, the aggressive management of traditional cardiovascular risk factors is essential. Risk reduction strategies and the management of these conditions, including hypertension hyperlipidemia, and diabetes mellitus, are described above [31,32,33]. According to the European Society of Cardiology guidelines, patients who have received thoracic radiation should have annual visits with a focused cardiovascular history and physical exam [41]. Symptoms to pay extra attention to include exercise intolerance, angina, shortness of breath, orthopnea, paroxysmal nocturnal dyspnea, palpitations, and syncope [41]. Physical exam findings include hypertension, elevated jugular venous pressure, pulmonary edema, lower extremity edema, orthopnea, and new murmurs. An annual ECG should also be performed as many conduction diseases are asymptomatic [41].

For patients who have symptoms of concern for cardiovascular disease, further testing should be completed. Although there is limited research to guide imaging modality selection and screening intervals, an understanding of the relative strengths and weaknesses of the available modalities should be used to guide clinicians. The European Association of Cardiovascular Imaging and the American Society of Echocardiography recommend TTE as the first diagnostic test for most asymptomatic patients [42]. Other testing commonly recommended for post-radiation cardiovascular surveillance includes coronary computed tomography (CT) and cardiac magnetic resonance imaging (MRI).

**Figure 2:**
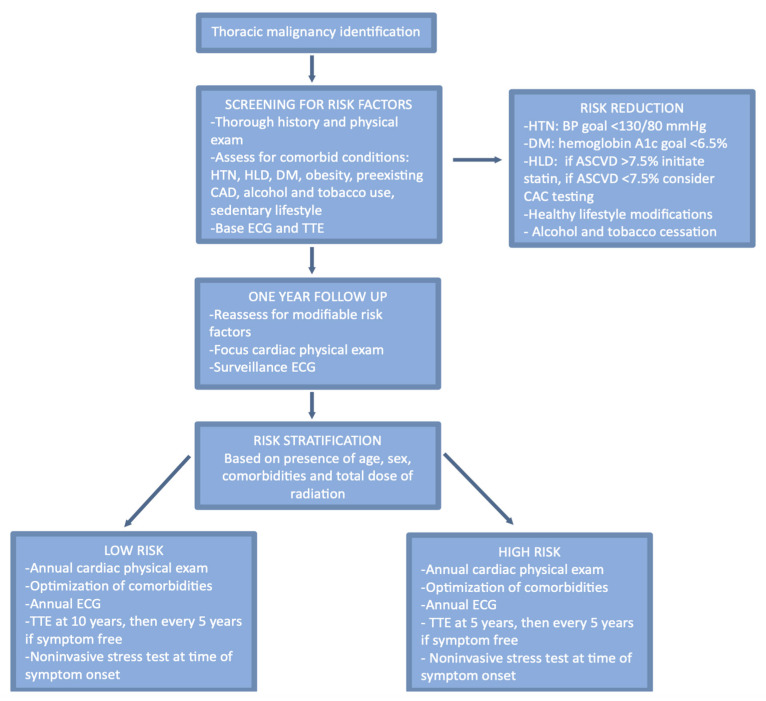
Algorithm for cardiovascular complication surveillance post mediastinal radiation therapy.

For asymptomatic patients, we recommend surveillance testing based on the patient’s overall risk assessment based on their pre-existing conditions and the details of their radiation therapy. High-risk patients have been defined as those with any of the following risk factors: a high cumulative dose (>30 Gy), younger patients (<50 years), higher doses of radiation fractions (>2Gy/day), presence and extent of the tumor in or next to the heart, lack of shielding, concomitant chemotherapy, cardiovascular risk factors, and pre-existing cardiovascular disease [42]. In addition to an annual visit and an ECG, we recommend surveillance echocardiography starting 5 years after the completion of radiation for high-risk patients and starting at 10 years for those considered low-risk (Figure 2) [42]. Functional stress testing to evaluate cardiac ischemia has also been recommended every 5 years beginning 5–10 years after therapy [42]. If all results of noninvasive testing are normal in a symptomatic patient, this requires further investigation. A right heart catheterization performed at rest and with exercise can accurately assess contractile reserve and may be able to aid in the early detection of radiation-induced cardiovascular disease.

## 8. Pre-Surgical Risk Stratification

Special considerations should be made in regards to preoperative risk stratification for patients undergoing planned cardiothoracic surgeries. Cardiothoracic surgery for patients who have received thoracic radiation therapy is associated with a higher mortality than those without radiation exposure [43]. It is recommended that any patient who has had radiation exposure at least 5 years prior to a planned cardiothoracic surgery should undergo a cardiovascular screening with an exercise stress test or echocardiogram, even if they are asymptomatic. A gated CT scan should be obtained to assess for mediastinal fibrosis, aortic calcifications, or other complications to guide surgical risk assessment and technique [43]. These recommendations are intended for risk stratification in patients undergoing high-risk surgeries.

## 9. Conclusions

Advancements in radiation oncology have led to increased awareness surrounding the cardiovascular side effects of mediastinal radiation therapy.

Minimizing the risks of radiation-induced cardiovascular disease should begin with the pre-radiation screening and optimization of pre-existing diseases as outlined in this article. Additionally, risk-reducing strategies should be used during radiation treatments to minimize the heart’s exposure to radiation.

Current research supports routine surveillance for patients who received thoracic radiation. The early detection of cardiovascular disease is associated with better outcomes.

The aim of this article is to summarize the known radiation-induced cardiotoxicity effects and to provide an easily accessible summary of guideline-driven screening recommendations for clinicians. These recommendations have been summarized and compiled from the referenced national society guidelines.

Continuing the inter-specialty collaboration among cardiologists, oncologists, and radiation oncologists to further advance the prevention and management of radiation-induced cardiotoxicity is vital. Like many facets of medicine, multi-disciplinary care has been shown to provide better patient outcomes. While this paper provides an adequate summary, we hope it also serves to demonstrate areas that still require further research. Given the vast advancements in radiation oncology, it is more important than ever to continue research into the prevention and management of the long-term sequelae of these therapies.

## Data Availability

Not applicable.

## References

[1] Ramin C., Schaeffer M.L., Zheng Z., Connor A.E., Hoffman-Bolton J., Lau B., Visvanathan K. (2021). All-Cause and Cardiovascular Disease Mortality among Breast Cancer Survivors in CLUE II, a Long-Standing Community-Based Cohort. JNCI J. Natl. Cancer Inst..

[2] Groarke J.D., Nguyen P.L., Nohria A., Ferrari R., Cheng S., Moslehi J. (2013). Cardiovascular complications of radiation therapy for thoracic malignancies: The role for non-invasive imaging for detection of cardiovascular disease. Eur. Hear. J..

[3] Liu X.-C., Zhou P.-K. (2022). Tissue Reactions and Mechanism in Cardiovascular Diseases Induced by Radiation. Int. J. Mol. Sci..

[4] Belzile-Dugas E., Eisenberg M.J. (2021). Radiation-induced cardiovascular disease: Review of an underrecognized pathology. J. Am. Heart Assoc..

[5] Darby S.C., Ewertz M., McGale P., Bennet A.M., Blom-Goldman U., Brønnum D., Correa C., Cutter D., Gagliardi G., Gigante B. (2013). Risk of Ischemic Heart Disease in Women after Radiotherapy for Breast Cancer. N. Engl. J. Med..

[6] Khalid Y., Fradley M., Dasu N., Dasu K., Shah A., Levine A. (2020). Gender disparity in cardiovascular mortality following radiation therapy for Hodgkin’s lymphoma: A systematic review. Cardio-Oncol..

[7] Chang H.-M., Okwuosa T.M., Scarabelli T., Moudgil R., Yeh E.T. (2017). Cardiovascular Complications of Cancer Therapy: Best Practices in Diagnosis, Prevention, and Management: Part 2. J. Am. Coll. Cardiol..

[8] Fukada J., Shigematsu N., Takeuchi H., Ohashi T., Saikawa Y., Takaishi H., Hanada T., Shiraishi Y., Kitagawa Y., Fukuda K. (2013). Symptomatic pericardial effusion after chemoradiation therapy in esophageal cancer patients. Int. J. Radiat. Oncol..

[9] Veinot J.P., Edwards W.D. (1996). Pathology of radiation-induced heart disease: A surgical and autopsy study of 27 cases. Hum. Pathol..

[10] Ghosh A.K., Crake T., Manisty C., Westwood M. (2018). Pericardial Disease in Cancer Patients. Curr. Treat. Options Cardiovasc. Med..

[11] Lee P.J., Mallik R. (2005). Cardiovascular Effects of Radiation Therapy: Practical Approach to Radiation Therapy-Induced Heart Disease. Cardiol. Rev..

[12] Marinko T. (2018). Pericardial disease after breast cancer radiotherapy. Radiol. Oncol..

[13] Imazio M., Andreis A., Agosti A., Piroli F., Avondo S., Casula M., Paneva E., Squarotti G.B., Giustetto C., De Ferrari G.M. (2021). Usefulness of Beta-blockers to Control Symptoms in Patients with Pericarditis. Am. J. Cardiol..

[14] Adler Y., Charron P., Imazio M., Badano L., Barón-Esquivias G., Bogaert J., Brucato A., Gueret P., Klingel K., Lioni C. (2015). ESC Guidelines for the diagnosis and management of pericardial diseases. Eur. Heart J..

[15] Seifert F.C., Miller D.C., Oesterle S.N., Oyer P.E., Stinson E.B., Shumway N.E. (1985). Surgical treatment of constrictive pericarditis: Analysis of outcome and diagnostic error. Circulation.

[16] Bertog S.C., Thambidorai S.K., Parakh K., Schoenhagen P., Ozduran V., Houghtaling P.L., Lytle B.W., Blackstone E.H., Lauer M.S., Klein A.L. (2004). Constrictive pericarditis: Etiology and cause-specific survival after pericardiectomy. J. Am. Coll. Cardiol..

[17] Hull M.C., Morris C.G., Pepine C.J., Mendenhall N.P. (2003). Valvular Dysfunction and Carotid, Subclavian, and Coronary Artery Disease in Survivors of Hodgkin Lymphoma Treated With Radiation Therapy. JAMA.

[18] Heidenreich P.A., Hancock S.L., Vagelos R.H., Lee B.K., Schnittger I. (2005). Diastolic dysfunction after mediastinal irradiation. Am. Heart J..

[19] Wu W., Masri A., Popovic Z.B., Smedira N.G., Lytle B.W., Marwick T.H., Griffin B.P., Desai M.Y. (2013). Long-term survival of patients with radiation heart disease undergoing cardiac surgery. Circulation.

[20] Al-Kindi S.G., Oliveira G.H. (2016). Heart transplantation outcomes in radiation-induced restrictive cardiomyopathy. J. Card. Fail..

[21] Saxena P., Joyce L.D., Daly R.C., Kushwaha S.S., Schirger J.A., Rosedahl J., Dearani J.A., Kara T., Edwards B.S. (2014). Cardiac transplantation for radiation-induced cardiomyopathy: The mayo clinic experience. Ann. Thorac. Surg..

[22] Özyurt H., Çevik O., Özgen Z., Özden A.S., Çadırcı S., Elmas M.A., Ercan F., Gören M.Z., Şener G. (2014). Quercetin protects radiation-induced DNA damage and apoptosis in kidney and bladder tissues of rats. Free Radic. Res..

[23] Quagliariello V., Berretta M., Buccolo S., Iovine M., Paccone A., Cavalcanti E., Taibi R., Montopoli M., Botti G., Maurea N. (2021). Polydatin Reduces Cardiotoxicity and Enhances the Anticancer Effects of Sunitinib by Decreasing Pro-Oxidative Stress, Pro-Inflammatory Cytokines, and NLRP3 Inflammasome Expression. Front. Oncol..

[24] Cutter D.J., Schaapveld M., Darby S.C., Hauptmann M., van Nimwegen F.A., Krol A.D.G., Janus C.P.M., van Leeuwen F.E., Aleman B.M.P. (2015). Risk for valvular heart disease after treatment for Hodgkin lymphoma. JNCI J. Natl. Cancer Inst..

[25] Murbraech K., Wethal T., Smeland K.B., Holte H., Loge J.H., Holte E., Rösner A., Dalen H., Kiserud C.E., Aakhus S. (2016). Valvular Dysfunction in Lymphoma Survivors Treated With Autologous Stem Cell Transplantation: A National Cross-Sectional Study. JACC Cardiovasc. Imaging.

[26] Lee C., Hahn R.T. (2022). Valvular Heart Disease Associated With Radiation Therapy: A Contemporary Review. Struct. Heart.

[27] Zhang D., Guo W., Al-Hijji M.A., El Sabbagh A., Lewis B.R., Greason K., Sandhu G.S., Eleid M.F., Holmes D.R., Herrmann J. (2019). Outcomes of Patients With Severe Symptomatic Aortic Valve Stenosis After Chest Radiation: Transcatheter Versus Surgical Aortic Valve Replacement. J. Am. Heart Assoc..

[28] Scarfò I., Denti P., Citro R., Buzzatti N., Alfieri O., La Canna G. (2019). MitraClip for radiotherapy-related mitral valve regurgitation. Hell. J. Cardiol..

[29] Adams M., Hardenbergh P.H., Constine L.S., Lipshultz S.E. (2003). Radiation-associated cardiovascular disease. Crit. Rev. Oncol..

[30] Adams M.J., Lipsitz S.R., Colan S.D., Tarbell N.J., Treves S.T., Diller L., Greenbaum N., Mauch P., Lipshultz S.E. (2004). Cardiovascular status in long-term survivors of Hodgkin’s disease treated with chest radiotherapy. J. Clin. Oncol..

[31] Wu S., Xu Y., Zheng R., Lu J., Li M., Chen L., Huo Y., Xu M., Wang T., Zhao Z. (2022). Hypertension Defined by 2017 ACC/AHA Guideline, Ideal Cardiovascular Health Metrics, and Risk of Cardiovascular Disease: A Nationwide Prospective Cohort Study. Lancet Reg. Healt West. Pac..

[32] Mancia(Chairperson) G., Kreutz(Co-Chair) R., Brunström M., Burnier M., Grassi G., Januszewicz A., Muiesan M.L., Tsioufis K., Agabiti-Rosei E., Algharably E.A.E. (2023). 2023 ESH Guidelines for the management of arterial hypertension The Task Force for the management of arterial hypertension of the European Society of Hypertension Endorsed by the International Society of Hypertension (ISH) and the European Renal Association (ERA). J. Hypertens..

[33] Arnett D.K., Blumenthal R.S., Albert M.A., Buroker A.B., Goldberger Z.D., Hahn E.J., Himmelfarb C.D., Khera A., Lloyd-Jones D., McEvoy J.W. (2019). 2019 ACC/AHA Guideline on the Primary Prevention of Cardiovascular Disease: A Report of the American College of Cardiology/American Heart Association Task Force on Clinical Practice Guidelines. Circulation.

[34] American Diabetes Association (2010). Standards of medical care in diabetes—2010. Diabetes Care.

[35] Hooning M.J., Botma A., Aleman B.M.P., Baaijens M.H.A., Bartelink H., Klijn J.G.M., Taylor C.W., van Leeuwen F.E. (2007). Long-term risk of cardiovascular disease in 10-year survivors of breast cancer. JNCI J. Natl. Cancer Inst..

[36] Martel M.K., Sahijdak W.M., Haken R.K.T., Kessler M.L., Turrisi A.T. (1998). Fraction size and dose parameters related to the incidence of pericardial effusions. Int. J. Radiat. Oncol..

[37] Kepka L., Socha J. (2021). Dose and fractionation schedules in radiotherapy for non-small cell lung cancer. Transl. Lung Cancer Res..

[38] Lin S.H., Hobbs B.P., Verma V., Tidwell R.S., Smith G.L., Lei X., Corsini E.M., Mok I., Wei X., Yao L. (2020). Randomized Phase IIB Trial of Proton Beam Therapy Versus Intensity-Modulated Radiation Therapy for Locally Advanced Esophageal Cancer. J. Clin. Oncol..

[39] Simonetto C., Eidemüller M., Gaasch A., Pazos M., Schönecker S., Reitz D., Kääb S., Braun M., Harbeck N., Niyazi M. (2019). Does deep inspiration breath-hold prolong life? Individual risk estimates of ischaemic heart disease after breast cancer radiotherapy. Radiother. Oncol..

[40] Hancock S.L., Tucker M.A., Hoppe R.T. (1993). Factors Affecting Late Mortality From Heart Disease After Treatment of Hodgkin’s Disease. JAMA.

[41] Lyon A.R., López-Fernández T., Couch L.S., Asteggiano R., Aznar M.C., Bergler-Klein J., Boriani G., Cardinale D., Cordoba R., Cosyns B. (2022). ESC Scientific Document Group. 2022 ESC Guidelines on cardio-oncology developed in collaboration with the European He-matology Association (EHA), the European Society for Therapeutic Radiology and Oncology (ESTRO) and the International Cardio-Oncology Society (IC-OS): Developed by the task force on cardio-oncology of the European Society of Cardiology (ESC). Eur. Heart J..

[42] Lancellotti P., Nkomo V.T., Badano L.P., Bergler-Klein J., Bogaert J., Davin L., Cosyns B., Coucke P., Dulgheru R., Edvardsen T. (2013). European Society of Cardiology Working Groups on Nuclear Cardiology and Cardiac Computed Tomography and Cardio-vascular Magnetic Resonance; American Society of Nuclear Cardiology; Society for Cardiovascular Magnetic Resonance; Society of Cardiovascular Computed Tomography. Expert consensus for multi-modality imaging evaluation of cardiovascular com-plications of radiotherapy in adults: A report from the European Association of Cardiovascular Imaging and the American Society of Echocardiography. Eur. Heart J. Cardiovasc. Imaging.

[43] Murabito A., Hirsch E., Ghigo A. (2020). Mechanisms of Anthracycline-Induced Cardiotoxicity: Is Mitochondrial Dysfunction the Answer?. Front. Cardiovasc. Med..

